# Heat transfer augmentation in nanofluids via nanofins

**DOI:** 10.1186/1556-276X-6-154

**Published:** 2011-02-18

**Authors:** Peter Vadasz

**Affiliations:** 1 Department of Mechanical Engineering, Northern Arizona University, P. O. Box 15600, Flagstaff, AZ 86011-5600, USA; 2 Faculty of Engineering, University of KZ Natal, Durban 4041, South Africa

## Abstract

Theoretical results derived in this article are combined with experimental data to conclude that, while there is no improvement in the effective thermal conductivity of nanofluids beyond the Maxwell's effective medium theory (J.C. Maxwell, *Treatise on Electricity and Magnetism*, 1891), there is substantial heat transfer augmentation via nanofins. The latter are formed as attachments on the hot wire surface by yet an unknown mechanism, which could be related to electrophoresis, but there is no conclusive evidence yet to prove this proposed mechanism.

## Introduction

The impressive heat transfer enhancement revealed experimentally in nanofluid suspensions by Eastman et al. [1], Lee et al. [2], and Choi et al. [3] conflicts apparently with Maxwell's [4] classical theory of estimating the effective thermal conductivity of suspensions, including higher-order corrections and other than spherical particle geometries developed by Hamilton and Crosser [5], Jeffrey [6], Davis [7], Lu and Lin [8], Bonnecaze and Brady [9,10]. Further attempts for independent confirmation of the experimental results showed conflicting outcomes with some experiments, such as Das et al. [11] and Li and Peterson [12], confirming at least partially the results presented by Eastman et al. [1], Lee et al. [2], and Choi et al. [3], while others, such as Buongiorno and Venerus [13], Buongiorno et al. [14], show in contrast results that are in agreement with Maxwell's [4] effective medium theory. All these experiments were performed using the Transient-Hot-Wire (THW) experimental method. On the other hand, most experimental results that used optical methods, such as the "optical beam deflection" [15], "all-optical thermal lensing method" [16], and "forced Rayleigh scattering" [17] did not reveal any thermal conductivity enhancement beyond what is predicted by the effective medium theory. A variety of possible reasons for the excessive values of the effective thermal conductivity obtained in some experiments have been investigated, but only few succeeded to show a viable explanation. Jang and Choi [18] and Prasher et al. [19] show that convection due to Brownian motion may explain the enhancement of the effective thermal conductivity. However, if indeed this is the case then it is difficult to explain why this enhancement of the effective thermal conductivity is selective and is not obtained in all the nanofluid experiments. Alternatively, Vadasz et al. [20] showed that hyperbolic heat conduction also provides a viable explanation for the latter, although their further research and comparison with later-published experimental data presented by Vadasz and Govender [21] led them to discard this possibility.

Vadasz [22] derived theoretically a model for the heat conduction mechanisms of nanofluid suspensions including the effect of the surface area-to-volume ratio of the suspended nanoparticles/nanotubes on the heat transfer. The theoretical model was shown to provide a viable explanation for the excessive values of the effective thermal conductivity obtained experimentally [1-3]. The explanation is based on the fact that the THW experimental method used in all the nanofluid suspensions experiments listed above needs a major correction factor when applied to non-homogeneous systems. This time-dependent correction factor is of the same order of magnitude as the claimed enhancement of the effective thermal conductivity. However, no direct comparison to experiments was possible because the authors [1-3] did not report so far their temperature readings as a function of time, the base upon which the effective thermal conductivity is being evaluated. Nevertheless, in their article, Liu et al. [23] reveal three important new results that allow the comparison of Vadasz's [22] theoretical model with experiments. The first important new result presented by Liu et al. [23] is reflected in the fact that the value of "effective thermal conductivity" revealed experimentally using the THW method is time dependent. The second new result is that those authors present graphically their time-dependent "effective thermal conductivity" for three specimens and therefore allow the comparison of their results with the theoretical predictions of this study showing a very good fit as presented in this article. The third new result is that their time dependent "effective thermal conductivity" converges at steady state to values that according to our calculations confirm the validity of the classical Maxwell's theory [4] and its extensions [5-10].

The objective of this article is to provide an explanation that settles the conflict between the apparent enhancement of the effective thermal conductivity in some experiments and the lack of enhancement in other experiments. It is demonstrated that the transient heat conduction process in nanofluid suspensions produces results that fit well with the experimental data [23] and validates Maxwell's [4] method of estimating the effective thermal conductivity of suspensions. The theoretical results derived in this article are combined with experimental data [23] to conclude that, while there is no improvement in the effective thermal conductivity of nanofluids beyond the Maxwell's effective medium theory [4], there is nevertheless substantial heat transfer augmentation via nanofins. The latter are formed as attachments on the hot wire surface by a mechanism that could be related to electrophoresis and therefore such attachments depend on the electrical current passing through the wire, and varies therefore amongst different experiments. Also since the effective thermal conductivity does not increase beyond the Maxwell's [4] effective medium theory, the experiments using optical methods, such as Putnam et al. [15], Rusconi et al. [16] and Venerus et al. [17], are also consistent with the conclusion of this study.

In this article, a *contextual notation *is introduced to distinguish between dimensional and dimensionless variables and parameters. The *contextual notation *implies that an asterisk subscript is used to identify dimensional variables and parameters only when ambiguity may arise when the asterisk subscript is not used. For example t_* _is the dimensional time, while *t *is its corresponding dimensionless counterpart. However, *k*_f _is the effective fluid phase thermal conductivity, a dimensional parameter that appears without an asterisk subscript without causing ambiguity.

## Problem formulation

The theoretical model derived by Vadasz [22] to investigate the transient heat conduction in a fluid containing suspended solid particles by considering phase-averaged equations will be presented only briefly without including the details that can be obtained from [22]. The phase-averaged equations are

(1) $$\gamma_{\text{s}}\frac{\partial T_{\text{s}}}{\partial t_{*}}=h\left(T_{\text{f}}-T_{\text{s}}\right)$$

(2) $$\gamma_{\text{f}}\frac{\partial T_{\text{f}}}{\partial t_{*}}=k_{\text{f}}\nabla_{*}^{2}T_{\text{f}}-h\left(T_{\text{f}}-T_{\text{s}}\right)$$

where t_* _is time, *T*_f _(r_*_,t_*_), and *T*_s _(r_*_,t_*_) are temperature values for the fluid and solid phases, respectively, averaged over a representative elementary volume (REV) that is large enough to be statistically valid but sufficiently small compared to the size of the domain, and where r_* _are the coordinates of the centroid of the REV. In Equations (1) and (2), γ_s _= *ερ*_s_*c*_s _and γ_f _= (1 - *ε*)*ρ*_f_*c*_p _represent the effective heat capacity of the solid and fluid phases, respectively; with *ρ*_s _and *ρ*_f _are the densities of the solid and fluid phases, respectively; *c*_s _and *c*_p _are the specific heats of the solid and fluid phases, respectively; and *ε *is the volumetric solid fraction of the suspension. Similarly, *k*_f _is the effective thermal conductivity of the fluid that may be defined in the form $k_{\text{f}}=f(\varepsilon ,\kappa ){\tilde{k}}_{\text{f}}$, where ${\tilde{k}}_{\text{f}}$ is the thermal conductivity of the fluid, $\kappa ={\tilde{k}}_{\text{s}}/{\tilde{k}}_{\text{f}}$ is the thermal conductivity ratio, and *ε *is the solid fraction of suspended particles in the suspension. In Equations (1) and (2), the parameter *h*, carrying units of W m^-3 ^K^-1^, represents an integral heat transfer coefficient for the contribution of the heat conduction at the solid-fluid interface as a volumetric heat source/sink within an REV. It is assumed to be independent of time, and its general relationship to the surface-area-to-volume ratio (specific area) was derived in [22]. Note that *T*_s_(r_*_,t_*_) is a function of the space variables represented by the position vector $r_{*}=x_{*}{\overset{\wedge }{e}}_{x}+y_{*}{\overset{\wedge }{e}}_{y}+z_{*}{\overset{\wedge }{e}}_{z}$, in addition to its dependence on time, because *T*_s_(r_*_,t_*_) depends on *T*_f_(r_*_,t_*_) as explicitly stated in Equation (1), although no spatial derivatives appear in Equation (1). There is a lack of macroscopic level conduction mechanism in Equation (1) representing the heat transfer within the solid phase because the solid particles represent the dispersed phase in the fluid suspension, and therefore the solid particles can conduct heat between themselves only via the neighbouring fluid. When steady state is accomplished ∂*T*_s_/∂t_* _= ∂*T*_f_/∂t_* _= 0, leading to local thermal equilibrium between the solid and fluid phases, i.e. *T*_s_(***r***) = *T*_f_(***r***).

For the case of a thin hot wire embedded in a cylindrical container insulated on its top and bottom one can assume that the heat is transferred in the radial direction only, r_*_, rendering Equation (2) into

(3) $$\gamma_{\text{f}}\frac{\partial T_{\text{f}}}{\partial t_{*}}=k_{\text{f}}\frac{1}{r_{*}}\frac{\partial }{\partial r_{*}}\left(r_{*}\frac{\partial T_{\text{f}}}{\partial r_{*}}\right)-h\left(T_{\text{f}}-T_{\text{s}}\right)$$

In a homogeneous medium without solid-suspended particles, Equation (1) is not relevant and the last term in Equation (3) can also be omitted. The boundary and initial conditions applicable are an initial ambient constant temperature, *T*_C_, within the whole domain, an ambient constant temperature, *T*_C_, at the outer radius of the container and a constant heat flux, *q*_0_, over the fluid-wire interface that is related to the Joule heating of the wire in the form *q*_0 _= *iV*/(*πd*_w__*_l_*_), where *d*_w__* _and l_* _are the diameter and the length of the wire respectively, *i *is the electric current and *V *is the voltage drop across the wire. Vadasz [22] showed that the problem formulated by Equations (1) and (3) subject to appropriate initial and boundary conditions represents a particular case of Dual-Phase-Lagging heat conduction (see also [24-28]).

An essential component in the application of the THW method for estimating experimentally the effective thermal conductivity of the nanofluid suspension is the assumption that the nanofluid suspension behaves basically like a homogeneous material following Fourier law for the bulk. The THW method is well established as the most accurate, reliable and robust technique [29] for evaluating the thermal conductivity of fluids. A very thin (5-80 μm in diameter) platinum (alternatively tantalum) wire is embedded vertically in the selected fluid and serves as a heat source as well as a thermometer (see [22] for details). Because of the very small diameter and high thermal conductivity of the platinum wire, it can be regarded as a line heat source in an otherwise infinite cylindrical medium. The rate of heat generated per unit length (l_*_) of platinum wire due to a step change in voltage is therefore ${\dot{q}}_{l*}=iV/l_{*}$ W m^-1^. Solving for the radial heat conduction due to this line heat source leads to an approximated temperature solution in the wire's neighbourhood in the form

(4) $$T(r_{*},t_{*})\approx \frac{{\dot{q}}_{l*}}{4\pi k}\left[-\gamma_{0}+\ln\left(\frac{4\alpha t_{*}}{r_{*}^{2}}\right)\right]$$

provided a validity condition for the approximation is enforced, i.e. $t_{*}>>t_{0*}=r_{\text{w}*}^{2}/4\alpha$, where *r*_w* _is the radius of the platinum wire, $\alpha ={\tilde{k}}_{\text{f}}/\rho_{\text{f}}c_{\text{p}}$ is the fluid's thermal diffusivity, and *γ*_0 _= 0.5772156649 is Euler's constant. Equation (4) reveals a linear relationship, on a logarithmic time scale, between the temperature and time. Therefore, one way of evaluating the thermal conductivity is from the slope of this relationship evaluated at r_* _= *r*_w*_. For any two readings of temperature, *T*_1 _and *T*_2_, recorded at times *t*_1* _and *t*_2* _respectively, the thermal conductivity can be approximated using Equation (4) in the form:

(5) $$k\approx \frac{i\;V}{4\pi \left(T_{2}-T_{1}\right)l_{*}}\left[\ln\left(\frac{t_{2*}}{t_{1*}}\right)\right]$$

Equation (5) is a very accurate way of estimating the thermal conductivity as long as the validity condition is fulfilled. The validity condition implies the application of Equation (5) for long times only. However, when evaluating this condition to data used in the nanofluid suspensions experiments, one obtains that *t*_0* _~ 6 ms, and the time beyond which the solution (5) can be used reliably is therefore of the order of hundreds of milliseconds, not so long in the actual practical sense.

## Two methods of solution

While the THW method is well established for homogeneous fluids, its applicability to two-phase systems such as fluid suspensions is still under development, and no reliable validity conditions for the latter exist so far (see Vadasz [30] for a discussion and initial study on the latter). As a result, one needs to refer to the two-equation model presented by Equations (1) and (3), instead of the one Fourier type equation that is applicable to homogeneous media.

Two methods of solution are in principle available to solve the system of Equations (1) and (3). The first is the elimination method while the second is the eigenvectors method. By means of the elimination method, one may eliminate *T*_f _from Equation (1) in the form:

(6) $$T_{\text{f}}=\frac{\gamma_{\text{s}}}{h}\frac{\partial T_{\text{s}}}{\partial t_{*}}+T_{\text{s}}$$

and substitute it into Equation (3) hence rendering the two Equations (1) and (3), each of which depends on both *T*_s _and *T*_f_, into separate equations for *T*_s _and *T*_f_, respectively, in the form:

(7) $$\tau_{q}\frac{\partial^{2}T_{i}}{\partial t_{*}^{2}}+\frac{\partial T_{i}}{\partial t_{*}}=\alpha_{\text{e}}\left[\frac{1}{r_{*}}\frac{\partial }{\partial r_{*}}\left(r_{*}\frac{\partial T_{i}}{\partial r_{*}}\right)+\frac{\tau_{T}}{r_{*}}\frac{\partial }{\partial r_{*}}\left(r_{*}\frac{\partial^{2}T_{i}}{\partial r_{*}\partial t_{*}}\right)\right]\text{ for }i=\text{s},\text{f}$$

where the index *i *takes the values *i *= *s *for the solid phase and *i *= f for the fluid phase, and the following notation was used:

(8) $$\begin{array}{ccc} \tau_{\text{q}}=\frac{\gamma_{\text{s}}\gamma_{\text{f}}}{h\left(\gamma_{\text{s}}+\gamma_{\text{f}}\right)}; & \alpha_{\text{e}}=\frac{k_{\text{f}}}{\left(\gamma_{\text{s}}+\gamma_{\text{f}}\right)}; & \tau_{\text{T}}=\frac{\gamma_{\text{s}}k_{\text{f}}}{h\left(\gamma_{\text{s}}+\gamma_{\text{f}}\right)\alpha_{\text{e}}}=\frac{\gamma_{\text{s}}}{h} \end{array}$$

In Equation (8), τ_q _and τ_T _are the heat flux and temperature-related time lags linked to Dual-Phase-Lagging [22,24-27,31], while *α*_e _is the effective thermal diffusivity of the suspension. The resulting Equation (7) is identical for both fluid and solid phases. Vadasz [22] used this equation in providing the solution. The initial conditions applicable to the problem at hand are identical for both phases, i.e. both phases' temperatures are set to be equal to the ambient temperature *T*_C_

(9) $$t_{*}=0:\;T_{i}=T_{\text{C}}=\text{constant ,}\;\;\;\text{for}\;\;\;i=\text{s, f}$$

The boundary conditions are

(10) $$r_{*}=r_{0*}:\;\;T_{\text{f}}=T_{\text{C}}$$

(11) $$r_{*}=r_{\text{w}*}:\;\;{\left(\frac{\partial T_{\text{f}}}{\partial r_{*}}\right)}_{r_{*}=r_{\text{w}*}}=-\frac{q_{0}}{k_{\text{f}}}$$

where *r*_0* _is the radius of the cylindrical container. Equation (7) is second-order in time and second-order in space. The initial conditions (9) provide one such condition for each phase while the second-order Equation (7) requires two such conditions. To obtain the additional initial conditions, one may use Equations (1) and (3) in combination with (9). From (9), it is evident that both phases' initial temperatures at t_* _= 0 are identical and constant. Therefore, ${\left(T_{\text{f}}\right)}_{t_{*}=0}={\left(T_{\text{s}}\right)}_{t_{*}=0}=T_{\text{C}}=\text{constant}$, leading to ${\left(T_{\text{f}}-T_{\text{s}}\right)}_{t_{*}=0}=0$ and ${\left[\partial /\partial r_{*}\left(r_{*}\partial T_{\text{f}}/\partial r_{*}\right)\right]}_{t_{*}=0}=0$ to be substituted in (1) and (3), which in turn leads to the following additional initial conditions for each phase:

(12) $$t_{*}=0:\;{\left(\frac{\partial T_{i}}{\partial t_{*}}\right)}_{t_{*}=0}=0\;\;\;\;\text{for}\;\;\;i=s,f$$

The two boundary conditions (10) and (11) are sufficient to uniquely define the problem for the fluid phase; however, there are no boundary conditions set for the solid phase as the original Equation (1) for the solid phase had no spatial derivatives and did not require boundary conditions. To obtain the corresponding boundary conditions for the solid phase, which are required for the solution of Equation (7) corresponding to *i *= s, one may use first the fact that at r_* _= *r*_0* _both phases are exposed to the ambient temperature and therefore one may set

(13) $$r_{*}=r_{0*}:\;\;T_{\text{s}}=T_{\text{C}}$$

Second, one may use Equation (6) and taking its derivative with respect to r_* _yields

(14) $$\frac{\gamma_{\text{s}}}{h}\frac{\partial }{\partial t_{*}}\left(\frac{\partial T_{\text{s}}}{\partial r_{*}}\right)+\frac{\partial T_{\text{s}}}{\partial r_{*}}=\frac{\partial T_{\text{f}}}{\partial r_{*}}$$

In Equation (14), the spatial variable r_* _plays no active role; it may therefore be regarded as a parameter. As a result, one may present Equation (14) for any specified value of r_*_. Choosing r_* _= *r*_w* _where the value of ${\left(\partial T_{\text{f}}/\partial r_{*}\right)}_{r_{\text{w}*}}$ is known from the boundary condition (11), yields from (14) the following ordinary differential equation:

(15) $$\frac{\gamma_{\text{s}}}{h}\frac{\text{d}\;\;}{\text{d}t_{*}}{\left(\frac{\partial T_{s}}{\partial r_{*}}\right)}_{r_{\text{w}*}}+{\left(\frac{\partial T_{\text{s}}}{\partial r_{*}}\right)}_{r_{\text{w}*}}=-\frac{q_{0}}{k_{\text{f}}}$$

At steady state, Equation (15) produces the solution

(16) $${\left(\frac{\partial T_{\text{s},\text{st}}}{\partial r_{*}}\right)}_{r_{\text{w}*}}=-\frac{q_{0}}{k_{\text{f}}}$$

where *T*_s,st _is the steady-state solution. The transient solution *T*_s,tr _= *T*_s _- *T*_s,st _satisfies then the equation:

(17) $$\frac{\gamma_{\text{s}}}{h}\frac{\text{d}\;\;}{\text{d}t_{*}}{\left(\frac{\partial T_{\text{s,tr}}}{\partial r_{*}}\right)}_{r_{\text{w}*}}+{\left(\frac{\partial T_{\text{s,tr}}}{\partial r_{*}}\right)}_{r_{\text{w}*}}=0$$

subject to the initial condition

(18) $${\left[{\left(\frac{\partial T_{\text{s,tr}}}{\partial r_{*}}\right)}_{r_{\text{w}*}}\right]}_{t_{*}=0}={\left[{\left(\frac{\partial T_{\text{s}}}{\partial r_{*}}\right)}_{r_{\text{w}*}}\right]}_{t_{*}=0}=0$$

because ${\left[\partial T_{\text{s}}/\partial r_{*}\right]}_{t_{*}=0}=0$ for all values of $r_{*}\in \left[r_{\text{w}*},r_{\text{0}*}\right]$ given that according to (9) at t_* _= 0: ${\left(\;T_{\text{s}}\right)}_{t_{*}=0}={\left(T_{\text{f}}\right)}_{t_{*}=0}=T_{\text{C}}=\text{constant}$. Equation (17) can be integrated to yield

(19) $${\left(\frac{\partial T_{\text{s,tr}}}{\partial r_{*}}\right)}_{r_{\text{w}*}}=A\exp\left(-\frac{h}{\gamma_{\text{s}}}t\right)$$

which combined with the initial condition (18) produces the value of the integration constant *A *= 0 and therefore the transient solution becomes

(20) $${\left(\frac{\partial T_{s,tr}}{\partial r_{*}}\right)}_{r_{w*}}=0$$

The complete solution for the solid temperature gradient at the wire is therefore obtained by combining (20) with (16) leading to

(21) $${\left(\frac{\partial T_{\text{s}}}{\partial r_{*}}\right)}_{r_{\text{w}*}}=-\frac{q_{0}}{k_{\text{f}}}$$

producing the second boundary condition for the solid phase, which is identical to the corresponding boundary condition for the fluid phase. One may therefore conclude that the solution to the problem formulated in terms of Equation (7) that is identical to both phases, subject to initial conditions (9) and (12) that are identical to both phases, and boundary conditions (10), (11), and (13), (21) that are also identical to both phases, should be also identical to both phases, i.e. *T*_s _(t_*_,r_*_) = *T*_f _(t_*_,r_*_). This, however, may not happen because then *T*_f _- *T*_s _= 0 leads to conflicting results when substituted into (1) and (3). The result obtained here is identical to Vadasz [32] who demonstrated that a paradox revealed by Vadasz [33] can be avoided only by refraining from using this method of solution. While the paradox is revealed in the corresponding problem of a porous medium subject to a combination of Dirichlet and insulation boundary conditions, the latter may be applicable to fluids suspensions by setting the effective thermal conductivity of the solid phase to be zero. The fact that in the present case the boundary conditions differ, i.e. a constant heat flux is applied on one of the boundaries (such a boundary condition would have eliminated the paradox in porous media), does not eliminate the paradox in fluid suspensions mainly because in the latter case the steady-state solution is identical for both phases. In the porous media problem, the constant heat flux boundary condition leads to different solutions at steady state, and therefore the solutions for each phase even during the transient conditions differ.

The elimination method yields the same identical equation with identical boundary and initial conditions for both phases apparently leading to the wrong conclusion that the temperature of both phases should therefore be the same. A closer inspection shows that the discontinuity occurring on the boundaries' temperatures at *t *= 0, when a "ramp-type" of boundary condition is used, is the reason behind the occurring problem and the apparent paradox. The question that still remains is which phase temperature corresponds to the solution presented by Vadasz [22]; the fluid or the solid phase temperature?

By applying the eigenvectors method as presented by Vadasz [32], one may avoid the paradoxical solution and obtain both phases temperatures. The analytical solution to the problem using the eigenvectors method is obtained following the transformation of the equations into a dimensionless form by introducing the following dimensionless variables:

(22) $$q=\frac{q_{*}}{q_{0}},\;\theta_{i}=\frac{\left(T_{i}-T_{\text{C}}\right)k_{\text{f}}}{q_{0}r_{0*}},\;r=\frac{r_{*}}{r_{0*}},\;t=\frac{\alpha_{\text{e}}t_{*}}{r_{0*}^{2}}$$

where the following two dimensionless groups emerged:

(23) $${\text{Fo}}_{q}=\frac{\alpha_{\text{e}}\tau_{q}}{r_{0*}^{2}};{\text{ Fo}}_{T}=\frac{\alpha_{\text{e}}\tau_{T}}{r_{0*}^{2}}$$

representing a heat flux Fourier number and a temperature Fourier number, respectively. The ratio between them is identical to the ratio between the time lags, i.e.

(24) $$\beta =\frac{{\text{Fo}}_{T}}{{\text{Fo}}_{q}}=\frac{\tau_{T}}{\tau_{q}}=\frac{\gamma_{\text{s}}+\gamma_{\text{f}}}{\gamma_{\text{f}}}$$

Equations (1) and (3) expressed in a dimensionless form using the transformation listed above are

(25) $${\text{Fh}}_{\text{s}}\frac{\partial \theta_{\text{s}}}{\partial t}=\left(\theta_{\text{f}}-\theta_{\text{s}}\right)$$

(26) $${\text{Fh}}_{\text{f}}\frac{\partial \theta_{\text{f}}}{\partial t}=\frac{1}{{\text{Ni}}_{\text{f}}}\frac{1}{r}\frac{\partial }{\partial r}\left(r\frac{\partial \theta_{\text{f}}}{\partial r}\right)-\left(\theta_{\text{f}}-\theta_{\text{s}}\right)$$

where the following additional dimensionless groups emerged:

(27) $${\text{Fh}}_{\text{s}}=\frac{\alpha_{\text{e}}\gamma_{\text{s}}}{hr_{0*}^{2}}={\text{Fo}}_{T}=\frac{\gamma_{\text{s}}+\gamma_{\text{f}}}{\gamma_{\text{f}}}{\text{Fo}}_{q}=\beta {\text{Fo}}_{q}$$

(28) $${\text{Fh}}_{\text{f}}=\frac{\gamma_{\text{s}}+\gamma_{\text{f}}}{\gamma_{\text{s}}}{\text{Fo}}_{q}=\frac{\alpha_{\text{e}}\gamma_{\text{f}}}{hr_{0*}^{2}}=\frac{{\text{Fo}}_{T}}{\left(\beta -1\right)}=\frac{\beta }{\left(\beta -1\right)}{\text{Fo}}_{q}$$

(29) $${\text{Ni}}_{\text{f}}=\frac{hr_{0*}^{2}}{k_{\text{f}}}=\frac{\left(\beta -1\right)}{\beta^{2}{\text{Fo}}_{q}}$$

where Ni_f _is the fluid phase Nield number. The solutions to Equations (25) and (26) are subject to the following initial and boundary conditions obtained from (9), (10) and (11) transformed in a dimensionless form:

(30) $$t=0:\;\theta_{i}=0\;\;\;\text{for}\;\;\;i=\text{s,f}$$

The boundary conditions are

(31) $$r=1:\;\;\theta_{\text{f}}=0$$

(32) $$r=r_{\text{w}}:\;\;{\left(\frac{\partial \theta_{\text{f}}}{\partial r}\right)}_{r=r_{\text{w}}}=-1$$

No boundary conditions are required for *θ*_s_. The solution to the system of Equations (25)-(26) is obtained by a superposition of steady and transient solutions *θ*_*i*,st_(*r*) and *θ*_*i*,tr _(*t*,*r*), respectively, in the form:

(33) $$\theta_{i}\left(t,r\right)=\theta_{i,\text{st}}\left(r\right)+\theta_{i,\text{tr}}\left(t,r\right)\;\;\;\;\text{for}\;\;\;i=\text{s},\text{f}$$

Substituting (33) into (25)-(26) yields to the following equations for the steady state:

(34) $$\left(\theta_{\text{f,st}}-\theta_{\text{s,st}}\right)=0$$

(35) $$\frac{1}{{\text{Ni}}_{\text{f}}}\frac{1}{r}\frac{d\;}{dr}\left(r\frac{d\theta_{\text{f,st}}}{dr}\right)-\left(\theta_{\text{f,st}}-\theta_{\text{s,st}}\right)=0$$

leading to the following steady solutions which satisfy the boundary conditions (31) and (32):

(36) $$\theta_{\text{f,st}}\left(r\right)=\theta_{\text{s,st}}\left(r\right)=-r_{\text{w}}\ln r$$

The transient part of the solutions *θ*_*i*,tr _(*t*,*r*) can be obtained by using separation of variables leading to the following form of the complete solution:

(37) $$\theta_{i}=-r_{\text{w}}\ln r+\sum_{n=1}^{\infty }S_{\text{in}}\left(t\right)R_{\text{on}}\left(r\right)\;\text{ for}\;\;\;i=\text{s},\text{f}$$

Substituting (37) into (25)-(26) yields, due to the separation of variables, the following equation for the unknown functions *R*_on _(*r*):

(38) $$\frac{1}{r}\frac{d}{dr}\left(r\frac{dR_{\text{on}}}{dr}\right)+\kappa_{n}^{2}R_{\text{on}}=0$$

subject to the boundary conditions

(39) $$r=1:\;\;R_{\text{on}}=0$$

(40) $$r=r_{\text{w}}:\;\;{\left(\frac{dR_{\text{on}}}{dr}\right)}_{r=r_{\text{w}}}=0$$

and the following system of equations for the unknown functions *S*_in _(*t*), (*i = *s,f), i.e.

(41) $$\{\begin{array}{c} \frac{dS_{\text{s}n}}{dt}=aS_{\text{s}n}-aS_{\text{f}n}\\ \frac{dS_{\text{f}n}}{dt}=cS_{\text{s}n}+d_{n}S_{\text{f}n} \end{array}$$

where

(42) $$a=-{\text{Fh}}_{\text{s}}^{-\text{1}}=-\frac{1}{\beta {\text{Fo}}_{q}}\;;\;\;c={\text{Fh}}_{\text{f}}^{-1}=\frac{\left(\beta -1\right)}{\beta {\text{Fo}}_{q}};\;\;d_{n}=-\frac{\left(\kappa_{n}^{2}+{\text{Ni}}_{\text{f}}\right)}{{\text{Ni}}_{\text{f}}{\text{Fh}}_{\text{f}}}=-\beta \kappa_{n}^{2}-\frac{\left(\beta -1\right)}{\beta {\text{Fo}}_{q}}$$

and where the separation constant $\kappa_{n}^{2}$ represents the eigenvalues in space.

Equation (38) is the Bessel equation of order 0 producing solutions in the form of Bessel functions

(43) $$R_{\text{on}}\left(\kappa_{n},r\right)=Y_{0}\left(\kappa_{n}\right)J_{0}\left(\kappa_{n}r\right)-J_{0}\left(\kappa_{n}\right)Y_{0}\left(\kappa_{n}r\right)$$

Where *J*_0_(*κ*_*n*_*r*) and *Y*_0_(*κ*_*n*_*r*) are the order 0 Bessel functions of the first and second kind, respectively. The solution (43) satisfies the boundary condition (39) as can easily be observed by substituting *r *= 1 in (43). Imposing the second boundary condition (40) yields a transcendental equation for the eigenvalues *κ*_*n *_in the form:

(44) $$J_{0}\left(\kappa_{n}\right)Y_{1}\left(\kappa_{n}r_{\text{w}}\right)-Y_{0}\left(\kappa_{n}\right)J_{1}\left(\kappa_{n}r_{\text{w}}\right)=0$$

where *J*_1_(*κ*_*n*_*r*_w_) and *Y*_1_(*κ*_*n*_*r*_w_) are the order 1 Bessel functions of the first and second kind, respectively, evaluated at *r *= *r*_w_. The compete solution is obtained by substituting (43) into (37) and imposing the initial conditions (30) in the form

(45) $${\left(\theta_{i}\right)}_{t=0}=-r_{\text{w}}\ln r+\sum_{n=1}^{\infty }S_{\text{in}}\left(0\right)R_{\text{on}}\left(r\right)\;=0\text{ for}\;i=\text{s},\text{f}$$

At *t *= 0, both phases' temperatures are the same leading to the conclusion that

(46) $$S_{\text{s}n}\left(0\right)=S_{\text{f}n}\left(0\right)=S_{no}$$

Multiplying (45) by the orthogonal eigenfunction *R*_*om *_(*κ*_*m *_*,r*) with respect to the weight function *r *and integrating the result over the domain [*r*_w_,1], i.e. $\int_{r_{\text{w}}}^{1}\left(•)R_{om}(\kappa_{m},r\right)\;r\;dr$ yield

(47) $$r_{\text{w}}\int_{r_{\text{w}}}^{1}r\ln rR_{om}\left(\kappa_{m},r\right)dr=\sum_{n=1}^{\infty }S_{no}\int_{r_{\text{w}}}^{1}rR_{on}\left(\kappa_{n},r\right)R_{om}\left(\kappa_{m},r\right)dr$$

The integral on the right-hand side of (47) produces the following result due to the orthogonality conditions for Bessel functions:

(48) $$\int_{r_{\text{w}}}^{1}r\;R_{on}\left(\kappa_{n},r\right)R_{om}\left(\kappa_{m},r\right)dr=\{\begin{array}{cc} 0\; & \text{for}\;\;n\neq m\\ N\left(\kappa_{n}\right) & \text{for}\;\;n=m \end{array}$$

where the norm *N*(*κ*_*n*_) is evaluated in the form:

(49) $$N\left(\kappa_{n}\right)=\int_{r_{\text{w}}}^{1}rR_{on}^{2}\left(\kappa_{n},r\right)dr=\frac{2}{\pi^{2}}\frac{\left[J_{1}^{2}\left(\kappa_{n}r_{w}\right)-J_{0}^{2}\left(\kappa_{n}\right)\right]}{\kappa_{n}^{2}J_{1}^{2}\left(\kappa_{n}r_{w}\right)}$$

The integral on the left-hand side of (47) can be evaluated using integration by parts and the equation for the eigenvalues (44) to yield

(50) $$\int_{r_{\text{w}}}^{1}r\ln rR_{on}\left(\kappa_{n},r\right)dr=\frac{1}{\kappa_{n}^{2}}\left[J_{0}\left(\kappa_{n}\right)Y_{0}\left(\kappa_{n}r_{w}\right)-Y_{0}\left(\kappa_{n}\right)J_{0}\left(\kappa_{n}r_{w}\right)\right]$$

Substituting (48) and (50) into (47) yields the values of *S*_in _at *t *= 0, i.e. *S*_*no *_= *S*_*sn*_(0) = *S*_f*n*_(0)

$$\frac{r_{\text{w}}}{\kappa_{n}^{2}}\left[J_{0}\left(\kappa_{n}\right)Y_{0}\left(\kappa_{n}r_{\text{w}}\right)-Y_{0}\left(\kappa_{n}\right)J_{0}\left(\kappa_{n}r_{\text{w}}\right)\right]=S_{no}N\left(\kappa_{n}\right)$$

that need to be used as initial conditions for the solution of system (41)

(51) $$S_{no}=\frac{\pi^{2}r_{\text{w}}J_{1}^{2}\left(\kappa_{n}r_{\text{w}}\right)\left[J_{0}\left(\kappa_{n}\right)Y_{0}\left(\kappa_{n}r_{\text{w}}\right)-Y_{0}\left(\kappa_{n}\right)J_{0}\left(\kappa_{n}r_{\text{w}}\right)\right]}{2\left[J_{1}^{2}\left(\kappa_{n}r_{\text{w}}\right)-J_{0}^{2}\left(\kappa_{n}\right)\right]}$$

to produce the explicit solutions in time. With the initial conditions for *S*_in _evaluated (*i *= s,f), one may turn to solving system (41) that can be presented in the following vector form:

(52) $$\frac{dS_{n}}{dt}=AS_{n}$$

where the matrix *A *is explicitly defined by

(53) $$A=\left|\begin{array}{cc} a & -a\\ c & d_{n} \end{array}\right|$$

with the values of *a*,*c *and *d*_*n *_given by Equation (42), and the vector ***S***_***n ***_defined in the form ***S***_***n ***_= [*S*_*sn*_,*S*_*fn*_]^T^. The eigenvalues *λ*_*n *_corresponding to (52) are obtained as the roots of the following quadratic algebraic equation:

(54) $$\lambda_{n}^{2}-\left(a+d_{n}\right)\lambda_{n}+a\left(d_{n}+c\right)=0$$

leading to

$$\begin{array}{ccc} \lambda_{1n}=\frac{a+d_{n}}{2}+\frac{1}{2}\sqrt{{\left(a-d_{n}\right)}^{2}-4ac} & \text{and} & \lambda_{2n}=\frac{a+d_{n}}{2}-\frac{1}{2}\sqrt{{\left(a-d_{n}\right)}^{2}-4ac} \end{array}$$

which upon substituting *a*,*c *and *d*_*n *_from Equation (42) yields

(55) $$\lambda_{1n}=-\frac{\left(1+\beta {\text{Fo}}_{q}\kappa_{n}^{2}\right)}{2{\text{Fo}}_{q}}\left[1+\sqrt{1-\frac{4{\text{Fo}}_{q}\kappa_{n}^{2}}{{\left(1+\beta {\text{Fo}}_{q}\kappa_{n}^{2}\right)}^{2}}}\right]$$

(56) $$\lambda_{2n}=-\frac{\left(1+\beta {\text{Fo}}_{q}\kappa_{n}^{2}\right)}{2{\text{Fo}}_{q}}\left[1-\sqrt{1-\frac{4{\text{Fo}}_{q}\kappa_{n}^{2}}{{\left(1+\beta {\text{Fo}}_{q}\kappa_{n}^{2}\right)}^{2}}}\right]$$

The following useful relationship is obtained from (55) and (56):

(57) $$\lambda_{1n}\lambda_{2n}=\frac{\kappa_{n}^{2}}{{\text{Fo}}_{q}}$$

The corresponding eigenvectors *υ*_1__*n *_and *υ*_2__*n *_are evaluated in the form:

(58) $$\begin{array}{ccc} v_{1n}=\left[\frac{1}{\frac{\left(-\lambda_{1n}+a\right)}{a}}\right] & \text{and} & v_{1n}=\left[\frac{1}{\frac{\left(-\lambda_{2n}+a\right)}{a}}\right] \end{array}$$

leading to the following solution:

(59) $$S_{n}=v_{1n}C_{1n}e^{\lambda_{1n}t}+v_{2n}C_{2n}e^{\lambda_{2n}t}$$

and explicitly following the substitution of (58) and the initial conditions *S*_in _(*i *= s,f), at *t *= 0, i.e. *S*_*sn*_(0) = *S*_f*n*_(0) = *S*_*no *_with the values of *S*_*no *_given by Equation (51)

(60) $$S_{\text{s}n}=\frac{S_{no}}{\left(\lambda_{2n}-\lambda_{1n}\right)}\left[\lambda_{2n}e^{\lambda_{1n}t}-\lambda_{1n}e^{\lambda_{2n}t}\right]$$

(61) $$S_{fn}=\frac{S_{no}}{\left(\lambda_{2n}-\lambda_{1n}\right)}\left[\lambda_{2n}\left(1+\beta Fo_{q}\;\lambda_{1n}\right)e^{\lambda_{1n}t}-\lambda_{1n}\left(1+\beta Fo_{q}\;\lambda_{2n}\right)e^{\lambda_{2n}t}\right]$$

Substituting (57) into (60) and (61) and the latter into the complete solution (37) yields

(62) $$\theta_{\text{s}}=-r_{\text{w}}\ln r+\sum_{n=1}^{\infty }B_{n}\left[\lambda_{2n}e^{\lambda_{1n}t}-\lambda_{1n}e^{\lambda_{2n}t}\right]R_{\text{on}}\left(r\right)$$

(63) $$\theta_{\text{f}}=-r_{\text{w}}\ln r+\sum_{n=1}^{\infty }B_{n}\left[\left(\lambda_{2n}+\beta \kappa_{n}^{2}\right)e^{\lambda_{1n}t}-\left(\lambda_{1n}+\beta \kappa_{n}^{2}\right)e^{\lambda_{2n}t}\right]R_{\text{on}}\left(r\right)$$

where *B*_*n *_is

(64) $$B_{n}=\frac{S_{no}}{\left(\lambda_{2n}-\lambda_{1n}\right)}=\frac{\pi^{2}r_{\text{w}}J_{1}^{2}\left(\kappa_{n}r_{\text{w}}\right)\left[J_{0}\left(\kappa_{n}\right)Y_{0}\left(\kappa_{n}r_{\text{w}}\right)-Y_{0}\left(\kappa_{n}\right)J_{0}\left(\kappa_{n}r_{\text{w}}\right)\right]}{2\left(\lambda_{2n}-\lambda_{1n}\right)\left[J_{1}^{2}\left(\kappa_{n}r_{\text{w}}\right)-J_{0}^{2}\left(\kappa_{n}\right)\right]}$$

Comparing the solutions obtained above with the solution obtained by Vadasz [22] via the elimination method, one may conclude that the latter corresponds to the solid phase temperature *θ*_s_.

The Fourier solution is presented now to compare the solution obtained from the Dual-Phase-Lagging model to the former. The Fourier solution is the result obtained by solving the thermal diffusion equation

(65) $$\frac{1}{\beta }\frac{\partial \theta }{\partial t}=\frac{1}{r}\frac{\partial }{\partial r}\left(r\frac{\partial \theta }{\partial r}\right)$$

subject to the boundary and initial conditions

(66) t=0: θ=0

(67) r=1:  θ=0

(68) $$r=r_{\text{w}}:\;\;{\left(\frac{\partial \theta }{\partial r}\right)}_{r=r_{\text{w}}}=-1$$

where the same scaling as in Equation (22) was applied in transforming the equation into its dimensionless form, hence the reason for the coefficient 1/*β *in the equation. The Fourier solution for this problem has then the form [34]

(69) $$\theta =-r_{\text{w}}\ln r+\sum_{n=1}^{\infty }C_{n}e^{-\beta \kappa_{n}^{2}t}R_{\text{on}}\left(r\right)$$

where

(70) $$C_{n}=\frac{\pi^{2}r_{\text{w}}J_{1}^{2}\left(\kappa_{n}r_{\text{w}}\right)\left[J_{0}\left(\kappa_{n}\right)Y_{0}\left(\kappa_{n}r_{\text{w}}\right)-Y_{0}\left(\kappa_{n}\right)J_{0}\left(\kappa_{n}r_{\text{w}}\right)\right]}{2\left[J_{1}^{2}\left(\kappa_{n}r_{\text{w}}\right)-J_{0}^{2}\left(\kappa_{n}\right)\right]}=S_{no}$$

and the eigenvalues *κ*_*n *_are the solution of the same transcendental Equation (44) and the eigenfunctions *R*_on_(*r*) are also identical to the ones presented in Equation (43). The relationship between the Fourier coefficient *C*_*n *_and the Dual-Phase-Lagging model's coefficient *B*_*n *_is

(71) $$C_{n}=\left(\lambda_{2n}-\lambda_{1n}\right)B_{n}$$

## Correction of the THW results

When evaluating the thermal conductivity by applying the THW method and using Fourier law, one obtains for the effective thermal conductivity the following relationship [22]:

(72) $$k_{\text{f,app}}=\frac{q_{0}r_{0*}}{\left[T_{\text{w}}\left(t\right)-T_{\text{C}}\right]}\left[-r_{\text{w}}\ln\left(r_{\text{w}}\right)+f\left(t\right)\right]$$

where the temperature difference [*T*_w_(t) - *T*_C_] is represented by the recorded experimental data, and the value of the heat flux at the fluid-platinum-wire interface *q*_0 _is evaluated from the Joule heating of the hot wire. In Equation (72) $f\left(t\right)=\sum_{n=1}^{\infty }C_{n}R_{\text{on}}\left(r_{\text{w}}\right)\exp\left(-\kappa_{n}^{2}t\right)$, where the coefficient *C*_*n *_is defined by (70) and the eigenvalues *κ*_*n *_are defined by Equation (44). Note that the definition of *C*_*n *_here is different than in [22]. The results obtained from the application of Equation (72) fit extremely well the approximation used by the THW method via Equation (5) within the validity limits of the approximation (5). Therefore, the THW method is extremely accurate for homogeneous materials.

On the other hand, for non-homogeneous materials, by means of the solutions (62) and (63) applicable to fluid suspensions evaluated at *r *= *r*_w_, one obtains

(73) $$\left[T_{\text{sw}}-T_{\text{C}}\right]=\frac{q_{0*}r_{0*}}{k_{\text{f,act}}}\left[-r_{\text{w}}\ln\left(r_{\text{w}}\right)+g_{\text{s}}\left(t\right)\right]$$

(74) $$\left[T_{\text{fw}}-T_{\text{C}}\right]=\frac{q_{0*}r_{0*}}{k_{\text{f,act}}}\left[-r_{\text{w}}\ln\left(r_{\text{w}}\right)+g_{\text{f}}\left(t\right)\right]$$

where *k*_f,act _is the actual effective thermal conductivity, *T*_sw _(*t*) and *T*_fw _(*t*) are the solid and fluid phases temperatures "felt" by the wire at the points of contact with each phase, respectively, and the functions *g*_s _(*t*) and *g*_f _(*t*) obtained from the solutions (62) and (63) evaluated at *r *= *r*_w _take the form

(75) $$g_{\text{s}}\left(t\right)=\sum_{n=1}^{\infty }B_{n}R_{\text{on}}\left(r_{\text{w}}\right)\left[\lambda_{n2}\exp\left(\lambda_{n1}t\right)-\lambda_{n1}\exp\left(\lambda_{n2}t\right)\right]$$

(76) $$g_{\text{f}}\left(t\right)=\sum_{n=1}^{\infty }B_{n}R_{\text{on}}\left(r_{\text{w}}\right)\left[\left(\lambda_{n2}+\beta \kappa_{n}^{2}\right)\exp\left(\lambda_{n1}t\right)-\left(\lambda_{n1}+\beta \kappa_{n}^{2}\right)\exp\left(\lambda_{n2}t\right)\right]$$

When the wire is exposed partly to the fluid phase and partly to the solid phase, there is no justification in assuming that the wire temperature is uniform: on the contrary the wire temperature will vary between the regions exposed to the fluid and solid phases. Assuming that some solid nanoparticles are in contact with the wire in a way that they form approximately "solid rings" around the wire, then the "effective" wire temperature can be evaluated as electrical resistances in series. By defining the relative wire area covered by the solid nanoparticles as *a*_s _= *A*_s_/*A*_tot _= *A*_s_/2*πr*_w*_l_* _its corresponding wire area covered by the fluid is *a*_f _= *A*_f_/*A*_tot _= 1 - *a*_s_, then from the relationship between the electrical resistance and temperature accounting for electrical resistances connected in series, one obtains an expression for the effective wire temperature (i.e. the temperature that is evaluated using the wire's lumped electrical resistance in the THW Wheatstone bridge) *T*_w _in the form:

(77) $$\left[T_{\text{w}}-T_{\text{C}}\right]=a_{\text{s}}\left(T_{\text{sw}}-T_{\text{C}}\right)+\left(1-a_{\text{s}}\right)\left(T_{\text{fw}}-T_{\text{C}}\right)$$

Substituting (73) and (74) into (77) yields

(78) $$\left[T_{\text{w}}-T_{\text{C}}\right]=\frac{q_{0*}r_{0*}}{k_{\text{f,act}}}\left[-r_{\text{w}}\ln\left(r_{\text{w}}\right)+a_{\text{s}}g_{\text{s}}\left(t\right)+\left(1-a_{\text{s}}\right)g_{\text{f}}\left(t\right)\right]$$

One may then use (78) to evaluate the actual nanofluid's effective thermal conductivity *k*_f,act _from (78) in the form

(79) $$k_{\text{f,act}}=\frac{q_{0*}r_{0*}}{\left(T_{\text{w}}-T_{\text{C}}\right)}\left[-r_{\text{w}}\ln\left(r_{\text{w}}\right)+a_{\text{s}}g_{\text{s}}\left(t\right)+\left(1-a_{\text{s}}\right)g_{\text{f}}\left(t\right)\right]$$

When using the single phase Fourier solution (72) applicable for homogeneous materials to evaluate the effective thermal conductivity of non-homogeneous materials like nanofluid suspensions instead of using Equation (79), one obtains a value that differs from the actual one by a factor of

(80) $$\sigma =\frac{k_{\text{f,app}}}{k_{\text{f,act}}}=\frac{\left[-r_{\text{w}}\ln\left(r_{\text{w}}\right)+f\left(t\right)\right]}{\left[-r_{\text{w}}\ln\left(r_{\text{w}}\right)+a_{\text{s}}g_{\text{s}}\left(t\right)+\left(1-a_{\text{s}}\right)g_{\text{f}}\left(t\right)\right]}$$

where *k*_f,app _is the apparent effective thermal conductivity obtained from the single phase Fourier conduction solution while *k*_f,act _is the actual effective thermal conductivity that corresponds to data that follow a Dual-Phase-Lagging conduction according to the derivations presented above. The ratio between the two provides a correction factor for the deviation of the apparent effective thermal conductivity from the actual one. This correction factor when multiplied by the ratio $k_{\text{f,act}}/{\tilde{k}}_{\text{f}}$ produces the results for $\sigma (k_{\text{f,act}}/{\tilde{k}}_{\text{f}})=k_{\text{f,app}}/{\tilde{k}}_{\text{f}}$, where ${\tilde{k}}_{\text{f}}$ is the thermal conductivity of the base fluid without the suspended particles, and *k*_f,act _is the effective thermal conductivity evaluated using Maxwell's [4] theory, which for spherical particles can be expressed in the form:

(81) $$\frac{k_{\text{f,act}}}{{\tilde{k}}_{\text{f}}}=1+\frac{3\varepsilon \left(\kappa -1\right)}{\left(\kappa +2\right)-\varepsilon \left(\kappa -1\right)}$$

where *k*_f,act _is Maxwell's effective thermal conductivity, $\kappa ={\tilde{k}}_{\text{s}}/{\tilde{k}}_{\text{f}}$ is the ratio between the thermal conductivity of the solid phase and the thermal conductivity of the base fluid, and *ε *is the volumetric solid fraction of the suspension. Then, these results of $k_{\text{f,app}}/{\tilde{k}}_{\text{f}}$ can be compared with the experimental results presented by Liu et al. [23].

## Results and discussion

**Figure 1:**
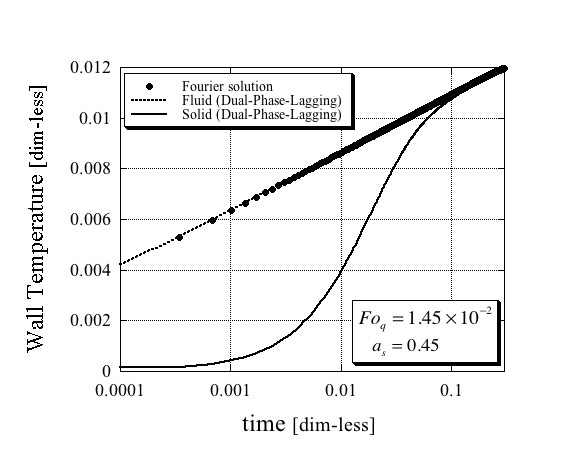
**Dimensionless wire temperature**. Comparison between the Fourier and Dual-Phase-Lagging solutions for the following dimensionless parameters values Fo_*q *_= 1.45 × 10^-2 ^and *a*_s _= 0.45.

**Figure 2:**
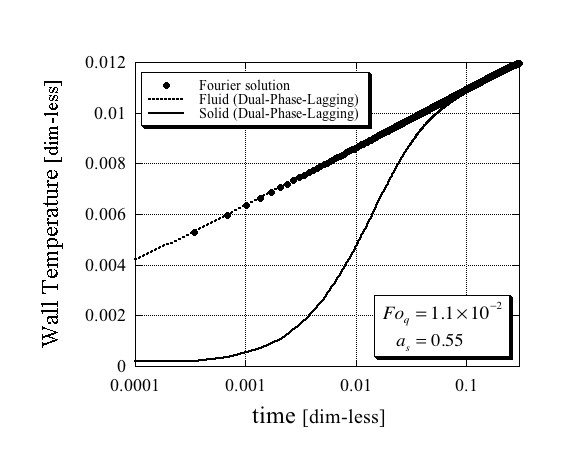
**Dimensionless wire temperature**. Comparison between the Fourier and Dual-Phase-Lagging solutions for the following dimensionless parameters values Fo_*q *_= 1.1 × 10^-2 ^and *a*_s _= 0.55.

**Figure 3:**
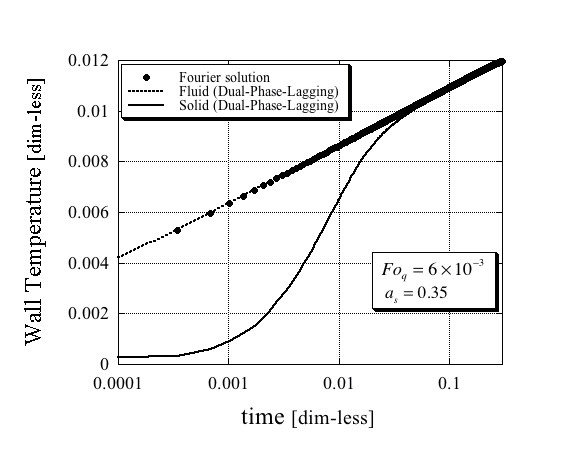
**Dimensionless wire temperature**. Comparison between the Fourier and Dual-Phase-Lagging solutions for the following dimensionless parameters values Fo_*q *_= 6 × 10^-3 ^and *a*_*s *_= 0.35.

The results for the solid and fluid phases' temperature at *r *= *r*_w _as a function of time obtained from the solutions (62) and (63) are presented in Figures 1, 2 and 3 in comparison with the single-phase Fourier solution (69) for three different combinations of values of Fo_*q *_and *a*_s_, and plotted on a logarithmic time scale. While the quantitative results differ amongst the three figures, there are some similar qualitative features that are important to mention. First, it is evident from these figures that the fluid phase temperature is almost the same as the temperature obtained from the single-phase Fourier solution. Second, it is also evident that the solid phase temperature lags behind the fluid phase temperature by a substantial difference. They become closer as steady-state conditions approach. It is therefore imperative to conclude that the only way, an excessively higher effective thermal conductivity of the nanofluid suspension as obtained by Eastman et al. [1], Lee et al. [2] and Choi et al. [3] could have been obtained even in an apparent form, is if the wire was excessively exposed to the solid phase temperature. The latter could have occurred if the electric current passing through the wire created electric fields that activated a possible mechanism of electrophoresis that attracted the suspended nanoparticles towards the wire. Note that such a mechanism does not cause agglomeration in the usual sense of the word, because as soon as the electric field ceases, the agglomeration does not have to persist and the particles can move freely from the wire's surface. Therefore, testing the wire's surface after such an experiment for evidence of agglomeration on the wire's surface may not necessarily produce the required evidence for the latter.

Liu et al. [23] used a very similar THW experimental method as the one used by Eastman et al. [1], Lee et al. [2] and Choi et al. [3] with the major distinction being in the method of producing the nanoparticles and a cylindrical container of different dimensions. They used water as the base fluid and Cu nanoparticles as the suspended elements at volumetric solid fractions of 0.1 and 0.2%. Their data that are relevant to the present discussion were digitized from their Figure 3[23] and used in the following presentation to compare our theoretical results. Three specimen data are presented in Figure 3[23] resulting in extensive overlap of the various curves, and therefore in some digitizing error which is difficult to estimate when using only this figure to capture the data.

**Figure 4:**
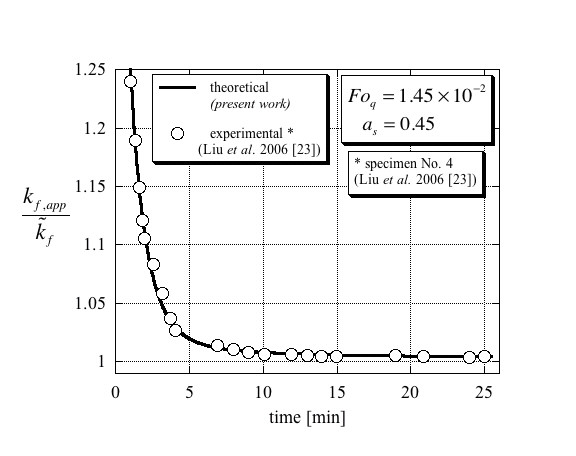
**Comparison of the present theory with experimental data of Liu et al. **[23] (here redrawn from published data) of the effective thermal conductivity ratio for conditions compatible with specimen No. 4, leading to a Fourier number of Fo_*q *_= 1.45 × 10^-2 ^and a solid particles to total wire area ratio of *a*_s _= 0.45.

**Figure 5:**
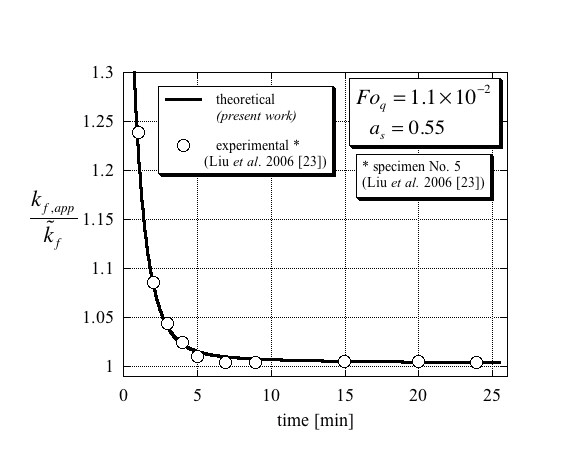
**Comparison of the present theory with experimental data of Liu et al. **[23] (here redrawn from published data) of the effective thermal conductivity ratio for conditions compatible with specimen No. 5, leading to a Fourier number of Fo_*q *_= 1.1 × 10^-2 ^and a solid particles to total wire area ratio of *a*_s _= 0.55.

**Figure 6:**
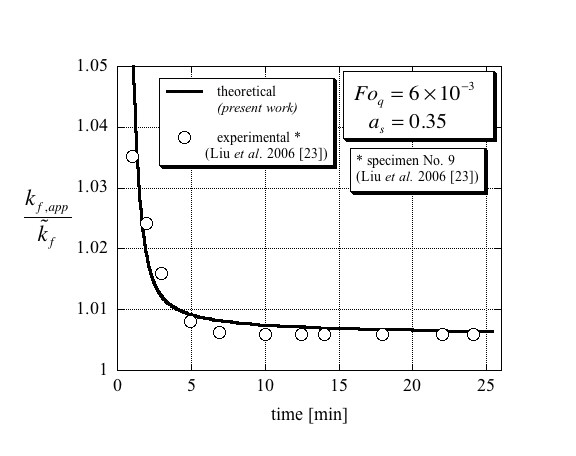
**Comparison of the present theory with experimental data of Liu et al. **[12] (here redrawn from published data) of the effective thermal conductivity ratio for conditions compatible with specimen No. 9, leading to a Fourier number of Fo_*q *_= 6 × 10^-3 ^and a solid particles to total wire area ratio of *a*_s _= 0.35.

The comparison between the theoretical results presented in this article with the experimental data [23] is presented in Figures 4, 5 and 6. The separation of these results into three different figures aims to better distinguish between the different curves and avoid overlapping as well as presenting the results on their appropriate scales. Figure 4 presents the results that are applicable to specimen No. 4 in Liu et al. [23] and corresponding to values of Fo_*q *_= 1.45 × 10^-2 ^and *a*_s _= 0.45 in the theoretical model. Evaluating Maxwell's [4] effective thermal conductivity for specimen No. 4 leads to a value of 0.6018 W/mK, which is higher by 0.3% than that of the base fluid (water), i.e. $k_{\text{f,act}}/{\tilde{k}}_{\text{f}}=1.003$. From the figure, it is evident that the theoretical results match very well with the digitized experimental data. Furthermore, the steady-state result for the ratio between the effective thermal conductivity and that of the base fluid was estimated from the digitized data to be $k_{\text{f,act}}/{\tilde{k}}_{\text{f}}=1.003\pm 0.001$ clearly validating Maxwell's [4] predicted value. The results applicable to specimen No. 5 in Liu et al. [23] and corresponding to values of Fo_*q *_= 1.1 × 10^-2 ^and *a*_*s *_= 0.55 in the theoretical model are presented in Figure 5. The very good match between the theory and the digitized experimental data is again evident. In addition, the ratio between the effective thermal conductivity and that of the base fluid was estimated from the digitized data to be $k_{\text{f,act}}/{\tilde{k}}_{\text{f}}=1.004\pm 0.001$ again validating Maxwell's [4] predicted value of $k_{\text{f,act}}/{\tilde{k}}_{\text{f}}=1.003$. The last result is presented in Figure 6, which corresponds to specimen No. 9 in Liu et al. [23] and to values of Fo_*q *_= 6 × 10^-3 ^and *a*_*s *_= 0.35 in the theoretical model. The results are presented on an appropriately scaled vertical axis and show again a very good match between the theory presented in this article, and the experimental data as digitized from Liu et al. [23]. Since the volumetric solid fraction for this specimen was 0.2%, its corresponding Maxwell's [4] effective thermal conductivity for this specimen leads to a value of 0.6036 W/mK, which is higher by 0.6% than that of the base fluid (water), i.e. $k_{\text{f,act}}/{\tilde{k}}_{\text{f}}=1.006$. The steady-state result for the ratio between the effective thermal conductivity and that of the base fluid was estimated from the digitized data to be $k_{\text{f,act}}/{\tilde{k}}_{\text{f}}=1.0059\pm 0.002$ validating again Maxwell's [4] predicted value.

It should be mentioned that Liu et al. [23] explain their time-dependent effective thermal conductivity by claiming that it was caused by nanoparticle agglomeration, a conclusion that is consistent with the theoretical results of this study.

## Conclusions

The theoretical results derived in this article combined with experimental data [23] lead to the conclusion that, while there is no improvement in the effective thermal conductivity of nanofluids beyond the Maxwell's effective medium theory [4], there is nevertheless the possibility of substantial heat transfer augmentation via nanofins. Nanoparticles attaching to the hot wire by a mechanism that could be related to electrophoresis depending on the strength of the electrical current passing through the wire suggests that such attachments can be deliberately designed and produced on any heat transfer surface to yield an agglomeration of nanofins that exchange heat effectively because of the extremely high heat transfer area as well as the flexibility of such nanofins to bend in the fluid's direction when fluid motion is present, hence extending its applicability to include a new, and what appears to be a very effective, type of heat convection. A quantitative estimate of the effectiveness of nanofins requires, however, an extension of the model presented in this article to include heat conduction within the nanofins.

## Abbreviations

REV: representative elementary volume; THW: transient-hot-wire.

## Competing interests

The author declares that they have no competing interests.

## Authors' contributions

PV conceived and carried out all work reported in this paper.
